# Evaluating genomic inbreeding of two Chinese yak (*Bos grunniens*) populations

**DOI:** 10.1186/s12864-024-10640-4

**Published:** 2024-07-24

**Authors:** Shi-Yi Chen, Zhihao Luo, Xianbo Jia, Junkun Zhou, Song-Jia Lai

**Affiliations:** Farm Animal Genetic Resources Exploration and Innovation Key Laboratory of Sichuan Province, Sichuan Agricultural University, Chengdu 211# Huimin Road, Wenjiang, Sichuan 611130 China

**Keywords:** Genetic diversity, Runs of homozygosity, ROH, Maiwa yaks, Jiulong yaks

## Abstract

**Background:**

Yaks are a vital livestock in the Qinghai-Tibetan Plateau area for providing food products, maintaining sustainable ecosystems, and promoting cultural heritage. Because of uncontrolled mating, it is impossible to estimate inbreeding level of yak populations using the pedigree-based approaches. With the aims to accurately evaluate inbreeding level of two Chinese yak populations (Maiwa and Jiulong), we obtained genome-wide single nucleotide polymorphisms (SNPs) by DNA sequencing and calculated five SNP-by-SNP estimators ($\:{F}_{HOM}$, $\:{F}_{L\&H}$, $\:{F}_{VR1}$, $\:{F}_{VR2}$, and $\:{F}_{YAN}$), as well as two segment-based estimators of runs of homozygosity (ROH, $\:{F}_{ROH}$) and homozygous-by-descent (HBD, $\:{F}_{HBD}$). Functional implications were analyzed for the positional candidate genes located within the related genomic regions.

**Results:**

A total of 151,675 and 190,955 high-quality SNPs were obtained from 71 Maiwa and 30 Jiulong yaks, respectively. Jiulong had greater genetic diversity than Maiwa in terms of allele frequency and nucleotide diversity. The two populations could be genetically distinguished by principal component analysis, with the mean differentiation index (Fst) of 0.0054. The greater genomic inbreeding levels of Maiwa yaks were consistently supported by all five SNP-by-SNP estimators. Based on simple proportion of homozygous SNPs ($\:{F}_{HOM}$), a lower inbreeding level was indicated by three successfully sequenced old leather samples that may represent historical Maiwa yaks about five generations ago. There were 3304 ROH detected among all samples, with mean and median length of 1.97 Mb and 1.0 Mb, respectively. A total of 94 HBD segments were found among all samples, whereas 92 of them belonged to the shortest class with the mean length of 10.9 Kb. Based on the estimates of $\:{F}_{ROH}$ and $\:{F}_{HBD}$, however, there was no difference in inbreeding level between Maiwa and Jiulong yaks. Within the genomic regions with the significant Fst or enriched by ROH, we found several candidate genes and pathways that have been reported to be related to diverse production traits in farm animals.

**Conclusions:**

We successfully evaluated the genomic inbreeding level of two Chinese yak populations. Although different estimators resulted in inconsistent conclusions on their genomic inbreeding levels, our results may be helpful to implement the genetic conservation and utilization programs for the two yak populations.

**Supplementary Information:**

The online version contains supplementary material available at 10.1186/s12864-024-10640-4.

## Background

Yaks (*Bos grunniens*) are a rare domestic bovine species and mainly geographically distributed in the Qinghai-Tibetan Plateau area [1]. Due to physiological adaptation to high-altitude environments [2], yaks play important roles in providing food products, maintaining sustainable ecosystems, and promoting cultural heritage. More than 90% of global yaks are distributed in China, and yak raising is a critical animal husbandry sector in several Chinese provinces, such as the Tibet, Qinghai, and Sichuan [3]. In contrast to the modern farming systems developed in dairy and beef cattle industry, yaks have been usually raised with more traditional practices, mainly the uncontrolled mating and grazing on natural pastures throughout the whole year. Because the mating can’t be recorded in such conditions, it is impossible to estimate the inbreeding level of yak populations using the pedigree-based approaches, which, however, is a critical parameter to effectively implement genetic conservation and utilization programs.

Individual inbreeding level has been often measured as the probability of two alleles at a given locus that are identical-by-descent, which is traditionally inferred from the pedigree relationships [4]. Due to the high-throughput capability to obtain a large number of genome-wide genetic markers during the past decades (mainly single nucleotide polymorphisms, SNPs), genetic marker-based inbreeding measures have been increasingly used with the greater precision and less bias compared with the pedigree-based approaches [5–8]. Methodologically, genetic marker-based inbreeding measures can be calculated either on the individual SNPs or by a large continuous chromosome segment, which were comprehensively compared by Caballero et al. [9]. One of obvious differences between the two types of inbreeding measures is that the SNP-by-SNP estimators always depend on the expected allele frequencies in some base population, which, therefore, is also termed variant frequency-based estimators. On the other hand, chromosome segment-based inbreeding measures lie in the fact that mating between two related individuals will produce the large homozygous chromosome segments (also termed runs of homozygosity, ROH) because of linkage disequilibrium (LD) among neighboring SNPs [10]. Therefore, the number and length pattern of ROH can be used for inferring the extent and time of inbreeding occurred. Another similar approach to measure the inbreeding is to detect large chromosome segments that are homozygous-by-descent (HBD) based on some specific statistical models [11, 12].

After the first publication of yak genome sequences in 2012 [2], genomic data have been popularly used for investigating genetic diversity and population structure [13–16], genome-wide association with production traits [17–19], and genetic basis of environmental adaptation [20–22] in yaks. To our best knowledge, few studies have yet been conducted to specially investigate the genomic inbreeding level in yaks. In southwest China, there are two well-known yak populations or breeds called Maiwa and Jiulong that are locally distributed in north-western plateau and western plateau of Sichuan province, respectively. The Jiulong yaks have greater body size than that of Maiwa, and their adult body weights of males could be up to 540 Kg and 320 Kg, respectively [19]. The estimated population size was ~ 1.6 million of Maiwa yaks in 2010 and ~ 40 thousand of Jiulong yaks in 2005 [23]. In this study, therefore, we obtained genome-wide SNPs using DNA sequencing approach and evaluated the genomic inbreeding of both Maiwa and Jiulong yaks, with the aims to: (1) reveal and compare genomic inbreeding level of the two yak populations, and (2) explore the functional implication of these positional candidate genes that are located within the significantly genetically differentiated and ROH-enriched genomic regions.

## Results

### SNPs, diversity and population structure

**Fig. 1:**
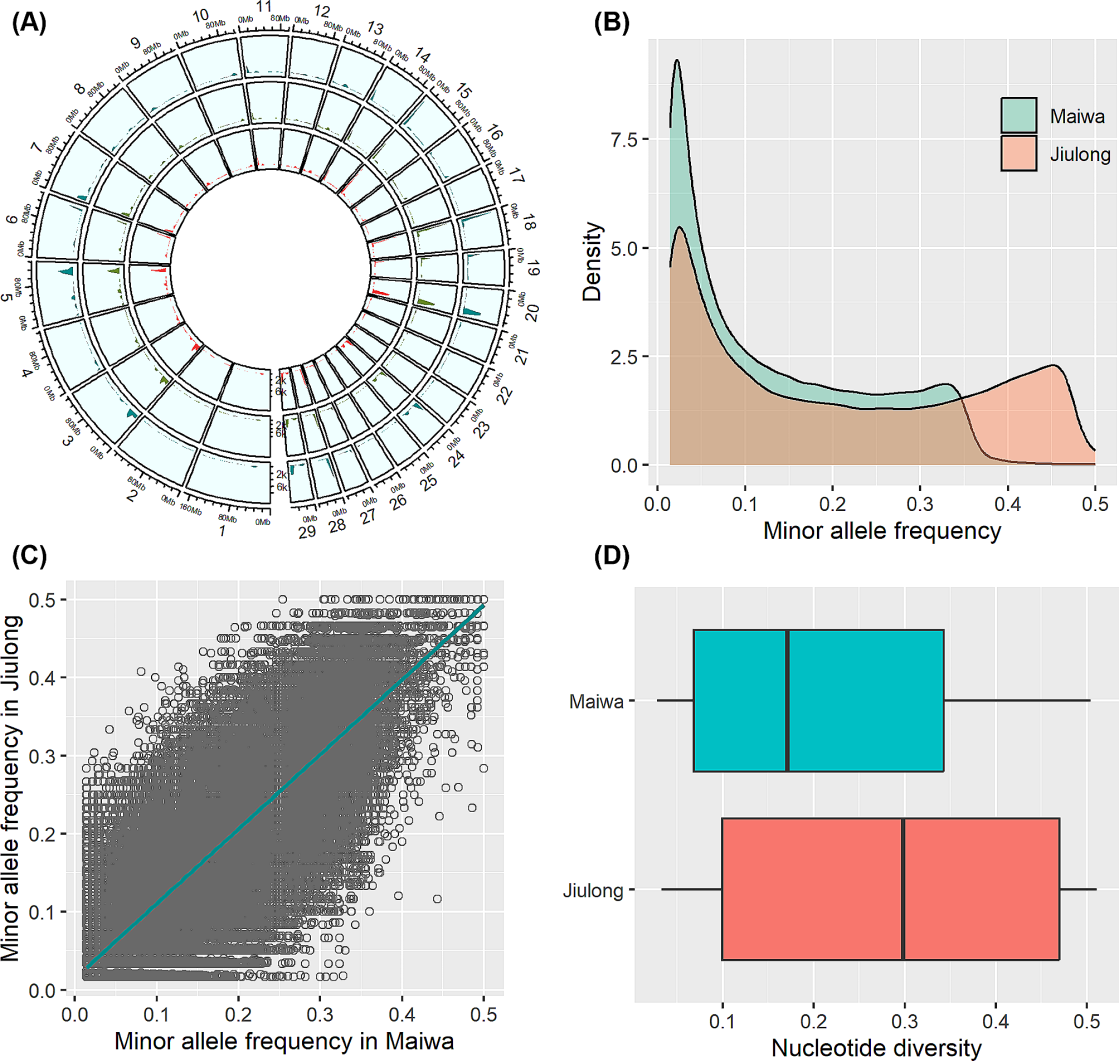
Genomic distribution and genetic diversity of SNPs. (**A**) The genomic distribution is shown for the SNPs found in Maiwa yaks (outside circle), Jiulong yaks (middle circle), and their common SNPs (inner circle). The distribution density of minor allele frequencies within each population and their pairwise correlations between Maiwa and Jiulong yaks are shown in (**B**) and (**C**), respectively. The nucleotide diversity of SNPs is shown by the boxplot (**D**)

The average number of clean paired-end sequencing reads per sample was 92.5, 91.3, and 90.3 million for Maiwa, Jiulong, and five old leather samples that may represent historical Maiwa yaks about five generations ago, respectively (Supplementary Table S1). A total of 29.3 million SNPs were initially obtained for the pooled samples of Maiwa and Jiulong yaks, in which 251,886 SNPs were retained for 71 Maiwa yaks and 30 Jiulong yaks after the quality controls. Further excluding SNPs with low minor allele frequency (MAF) and significant deviation from Hardy-Weinberg equilibrium (HWE), 151,675 and 190,955 clean SNPs were finally obtained for Maiwa and Jiulong yaks, respectively; 128,849 SNPs of them were overlapped between the two populations (Fig. 1A). The genome mapping rate ranged from 0.62 to 83.96% among the five old leather samples sequenced (Supplementary Table S1), by which 291,658 SNPs were initially obtained; however, 92.0% of these SNPs were private to single individual (Supplementary Figure S1). There were 24,133 SNPs that can be overlapped among Maiwa, Jiulong, and leather samples.

**Fig. 2:**
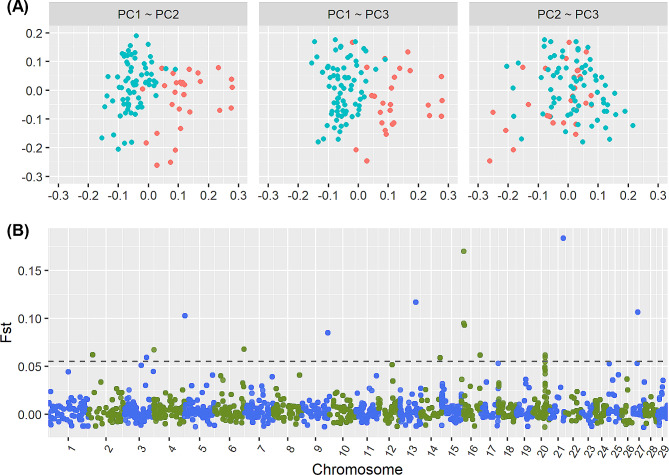
Sample clustering and population differentiation index Fst. (**A**) The top three principal components (PC1, PC2, and PC3) derived from the genome-wide SNPs are used for clustering Maiwa (blue) and Jiulong (red) yaks. The threshold of Fst is denoted by the horizontal dashed line in (**B**)

Across all genome-wide SNPs (Fig. 1B and C), average MAF in Maiwa yaks was lower than that in Jiulong yaks (0.131 vs. 0.207), which are consistent with the observed nucleotide diversity in Fig. 1D. The differences of MAF and nucleotide diversity between Maiwa and Jiulong yaks were further uniformly found on each chromosome (Supplementary Figure S2). After pruning out the tightly linked SNPs among the overlapped SNPs between Maiwa and Jiulong yaks, 106,853 SNPs were used for the principal component analysis (PCA), which indicated that individuals between Maiwa and Jiulong yaks, as well as within each population, could be clearly distinguished by the top three principal components (Fig. 2A), which explained 4.69%, 4.20%, and 3.93% of the total variance, respectively. The average genetic relatedness (± standard deviation, SD) among individuals calculated by the pruned SNP datasets were − 0.011 ± 0.014 and − 0.019 ± 0.013 for Maiwa and Jiulong yaks, respectively. The historical effective population sizes (Ne) estimated in Maiwa yaks were greater than that in Jiulong yaks (Supplementary Figure S3). Divided into 200 Kb chromosome windows, mean population differentiation index (Fst, ± SD) estimated was 0.0054 ± 0.0165 (Fig. 2B); and their distribution density was shown in Supplementary Figure S4. Based on the Fst threshold of 0.055 (i.e., mean + 3.5 SD), a total of 27 windows were suggested to be significant, which could be concatenated into 15 continuous genomic regions on 12 chromosomes (Table 1).

**Table 1:** Genomic regions and positional candidate genes revealed by the significant population differentiation index (fst) between Maiwa and Jiulong yaks

CHR	Positions (Mb)		Weighted Fst	Positional candiate genes
	Start	End		
Chr2	18.1	18.4	0.062	ENSBGRG00000018978, ENSBGRG00000019258, *DIS3L2*
Chr3	98.6	98.9	0.059	ENSBGRG00000000687
Chr4	0.2	0.4	0.067	ENSBGRG00000002406, ENSBGRG00000002460, ENSBGRG00000002499, ENSBGRG00000002506, *PRSS58*, ENSBGRG00000002524, ENSBGRG00000002539, ENSBGRG00000002555, *TRBV3-1*, ENSBGRG00000002580, ENSBGRG00000002588, ENSBGRG00000002605, ENSBGRG00000002616
Chr5	1.6	1.9	0.103	*CD69*, ENSBGRG00000018437, *KLRF1*, ENSBGRG00000018821, ENSBGRG00000019024, ENSBGRG00000019069, ENSBGRG00000019165, *KLRF2*, ENSBGRG00000019221
Chr6	126.6	126.9	0.068	*MARCHF1*, ENSBGRG00000005585
Chr9	112.1	112.4	0.085	*LPIN1*, *NTSR2*, *GREB1*, ENSBGRG00000024251
Chr13	68.1	68.4	0.117	*FDX1*, ENSBGRG00000012843, *RDX*
Chr14	82.9	83.2	0.059	*TMEM183A*, *PPFIA4*, *MYOG*, ENSBGRG00000026271, ENSBGRG00000026277, ENSBGRG00000026283, *ADORA1*, *MYBPH*, *CHI3L1*
Chr16	9.4	9.7	0.132	None
	12	12.3	0.093	DCHS2
	80.1	80.4	0.061	*SNAP29*, *CRKL*, *AIFM3*, *LZTR1*, *THAP7*, *LRRC74B*, *P2RX6*, ENSBGRG00000012728, *TUBA3D*, ENSBGRG00000013136, *SMPD4*, ENSBGRG00000018063, ENSBGRG00000018246, ENSBGRG00000018506, *KLHL22*, *SCARF2*
Chr20	51.7	51.9	0.062	*PPP6R1*, *TMEM86B*, ENSBGRG00000013851, ENSBGRG00000014020, *SYT5*, *TNNI3*, ENSBGRG00000015021, ENSBGRG00000015343, ENSBGRG00000015351, *EPS8L1*, ENSBGRG00000015460, *RDH13*, ENSBGRG00000015616
	52.1	52.3	0.059	ENSBGRG00000016803, ENSBGRG00000017049, ENSBGRG00000017092, ENSBGRG00000017267, ENSBGRG00000017302, ENSBGRG00000017313, ENSBGRG00000017516
Chr21	58.3	58.6	0.184	ENSBGRG00000016053, ENSBGRG00000016227, ENSBGRG00000016258
Chr27	19.8	20.1	0.106	None

CHR: chromosomes

### Genomic inbreeding

**Fig. 3:**
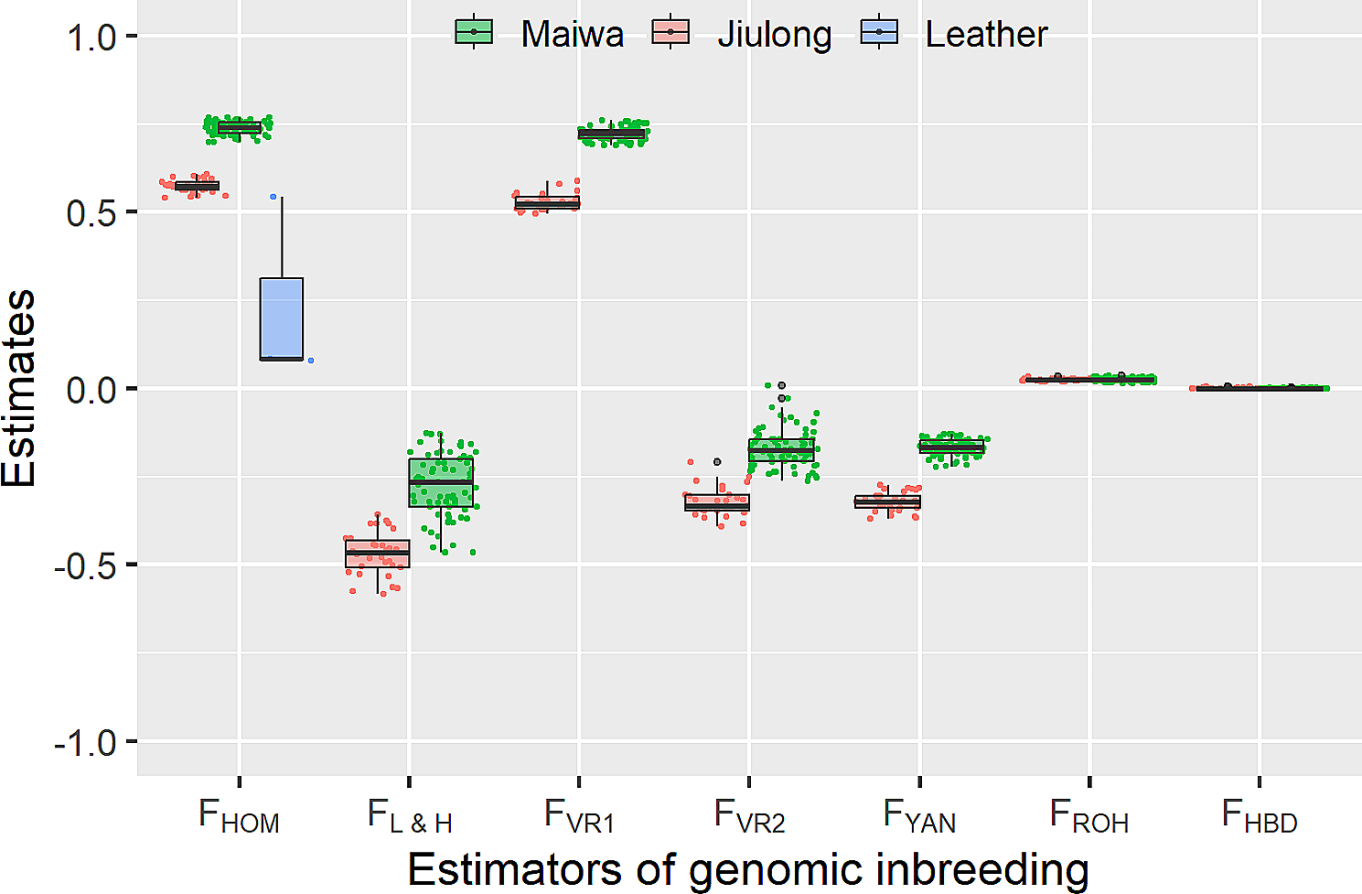
Estimates of genomic inbreeding estimators

The genomic inbreeding estimates were in Fig. 3. The greater inbreeding levels of Maiwa yaks than Jiulong yaks were consistently observed for the five SNP-by-SNP estimators. Small inter-individual variations were present within Maiwa or Jiulong population regarding all these five estimators. Among the four estimators that were adjusted by the allele frequencies observed in current populations, $\:{F}_{L\&H}$, $\:{F}_{VR2}$, and $\:{F}_{YAN}$ had the negative values in both populations, whereas $\:{F}_{VR1}$ ranged from 0.5 in Jiulong yaks to 0.8 in Maiwa yaks. The mean (± SD) of $\:{F}_{HOM}$, i.e., simple proportion of homozygous SNPs, was 0.738 ± 0.019 for Maiwa yaks, 0.573 ± 0.018 for Jiulong yaks, and 0.235 ± 0.266 for the leather samples. Pairwise Pearson correlation coefficients (r) among these SNP-by-SNP estimators were shown in Supplementary Figure S5; all of them were positive with the strongest and weakest correlations observed between $\:{F}_{HOM}$ and $\:{F}_{YAN}$ (*r* = 0.994, *P* < 0.001), and between $\:{F}_{L\&H}$ and $\:{F}_{VR2}$ (*r* = 0.442, *P* < 0.001), respectively.

**Fig. 4:**
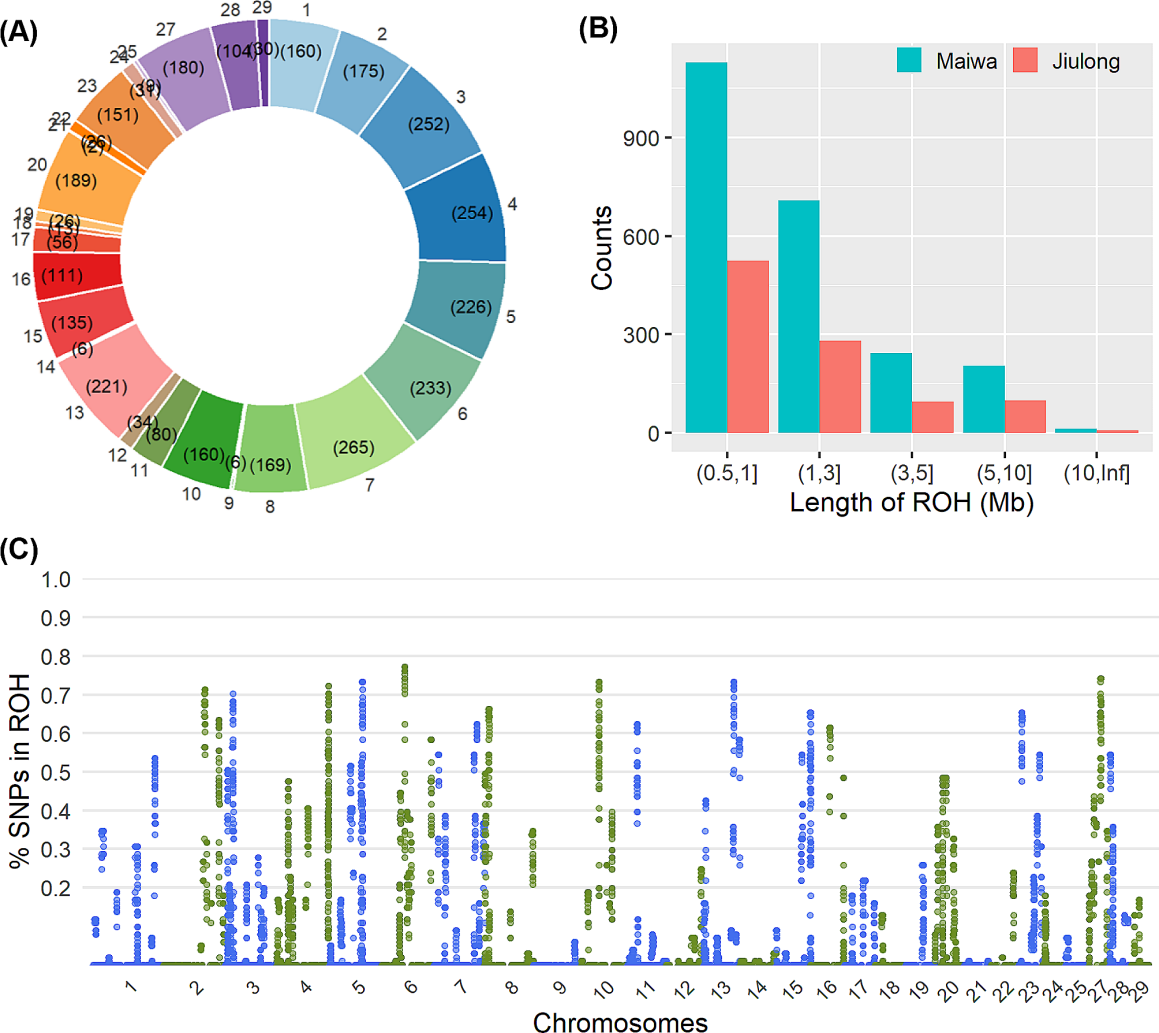
Numbers of runs of homozygosity (ROH) on each autosome (**A**), and their length distribution (**B**) and sample enrichment (**C**).

After excluding the tightly linked SNPs that were present in both populations, 184,302 SNPs were remained, by which a total of 3304 ROH were detected among all samples (Supplementary Table S2). The average number of ROH per individual was 32.4 for Maiwa yaks and 33.6 for Jiulong yaks, respectively. These ROH were located within 109 genomic regions and distributed among all autosomes with an exception of Chr26, with the highest number (*N* = 265) on Chr7 and lowest number (*N* = 2) on Chr21 (Fig. 4A). Among them, 16 and six genomic regions were uniquely found in Maiwa and Jiulong yaks, respectively; every one of them, however, was exclusively observed in one or two individuals. The mean and median of ROH length were 1.97 Mb and 1.0 Mb, respectively; and the length distributions between Maiwa and Jiulong populations were shown in Fig. 4B. There were 85 SNPs within the identified ROH that had been simultaneously found in more than 70% of individuals (Fig. 4C); the 100 Kb upstream and downstream of these SNPs could be concatenated into 15 continuous genomic regions (Table 2). The mean (± SD) of $\:{F}_{ROH}$ was 0.024 ± 0.005 for Maiwa yaks and 0.025 ± 0.003 for Jiulong yaks, respectively (Fig. 3); therefore, no difference of genomic inbreeding between the two populations was suggested by the ROH-based estimates.

**Table 2:** Functional enrichment results of positional candidate genes within genomic regions revealed by population differentiation index (Fst) or enriched by runs of homozygosity (ROH)

Source	Term name	Term id	Term size	adjusted *P*
*Region: Chr4:0.2–0.4 Mb*,* revealed by Fst*				
GO: MF	serine-type endopeptidase activity	GO:0004252	167	2.80E-05
GO: MF	serine-type peptidase activity	GO:0008236	181	3.80E-05
GO: MF	serine hydrolase activity	GO:0017171	184	4.10E-05
GO: MF	endopeptidase activity	GO:0004175	374	6.90E-04
GO: MF	peptidase activity	GO:0008233	556	3.30E-03
GO: MF	hydrolase activity	GO:0016787	1900	2.20E-02
*Region: Chr16:80.1–80.4*,* revealed by Fst*				
GO: MF	protein-macromolecule adaptor activity	GO:0030674	551	1.20E-02
GO: CC	Cul3-RING ubiquitin ligase complex	GO:0031463	26	1.50E-02
GO: MF	molecular adaptor activity	GO:0060090	623	2.20E-02
GO: CC	mediator complex	GO:0016592	33	2.40E-02
*Region: Chr3:21.8–22.0 Mb*,* enriched by ROH*				
GO: MF	methanethiol oxidase activity	GO:0018549	1	5.00E-02
*Region: Chr5:80.0-80.2 Mb*,* enriched by ROH*				
GO: CC	keratin filament	GO:0045095	77	3.90E-17
GO: CC	intermediate filament	GO:0005882	105	5.20E-16
GO: CC	intermediate filament cytoskeleton	GO:0045111	136	4.40E-15
GO: CC	polymeric cytoskeletal fiber	GO:0099513	359	1.20E-11
GO: CC	supramolecular fiber	GO:0099512	486	1.40E-10
GO: CC	supramolecular polymer	GO:0099081	493	1.50E-10
GO: CC	supramolecular complex	GO:0099080	708	2.80E-09
GO: CC	cytoskeleton	GO:0005856	1435	8.10E-07
GO: CC	intracellular non-membrane-bounded organelle	GO:0043232	3909	2.50E-03
GO: CC	non-membrane-bounded organelle	GO:0043228	3909	2.50E-03
GO: CC	cornified envelope	GO:0001533	24	6.60E-03
GO: BP	intermediate filament cytoskeleton organization	GO:0045104	40	8.30E-03
GO: BP	intermediate filament-based process	GO:0045103	41	8.80E-03
*Region: Chr5:81.0-81.2 Mb*,* enriched by ROH*				
GO: BP	viral gene expression	GO:0019080	55	3.00E-04
GO: BP	negative regulation of defense response	GO:0050687	12	3.80E-03
GO: MF	enzyme binding	GO:0019899	1352	4.20E-03
GO: BP	viral process	GO:0016032	219	1.90E-02
GO: BP	negative regulation of cytoplasmic pattern recognition receptor	GO:0039532	32	2.90E-02
GO: BP	viral transcription	GO:0019083	40	4.50E-02
GO: BP	regulation of defense response to virus	GO:0050688	41	4.70E-02

The genomic inbreeding estimated by seven HBD classes was in Supplementary Table S3. We found that the large proportions of inbreeding (mean = 67.9% and median = 99.2%) were derived from the shortest HBD segments. The mean of the summed genomic inbreeding across all HBD classes ($\:{F}_{HBD}$) was 0.038% (Maximum = 0.252% and SD = 0.048%) and 0.067% (Maximum = 0.510% and SD = 0.116%) for Maiwa and Jiulong yaks, respectively (Fig. 3). A total of 94 HBD segments were found among all Maiwa and Jiulong yaks, whereas 92 of them belonged to the shortest HBD class with mean length of 10.9 Kb. The remaining two HBD segments found in two individuals could be concatenated into one continuous genomic region (Chr27:6,984,748–23,946,830 bp).

### Functional implications of candidate genomic regions

There were 79 protein-encoding and five long non-coding RNA (lncRNA) positional genes located within the 15 genomic regions revealed by the significant Fst (Table 1 and Supplementary Table S4). Among them, several genes have been previously reported in literature to be significantly associated with various production and health traits in cattle, including the genes of lipin 1 (*LPIN1*), myogenin (*MYOG*), growth regulating estrogen receptor binding 1 (*GREB1*), myosin binding protein H (*MYBPH*), and troponin I3 cardiac type (*TNNI3*). Functional enrichment analysis revealed that the positional candidate genes located within the genomic region of Chr4:0.2–0.4 Mb were significantly enriched into six Gene Ontology (GO) terms of molecular functions (GO: MF), genes within the genomic region of Chr16:80.1–80.4 significantly enriched into two GO terms of cellular component (GO: CC) and two GO: MF terms (Table 2). Within the 15 genomic regions that were enriched by ROH, we found 33 protein-encoding and one lncRNA positional genes (Table 3 and Supplementary Table S5). These positional genes located within the genomic regions of Chr3:21.8–22.0 Mb, Chr5:80.0-80.2 Mb, and Chr5:81.0-81.2 Mb were significantly enriched into eight GO terms of biological process (GO: BP), 11 GO: CC, and two GO: MF (Table 2).

**Table 3:** Genomic regions and positional candidate genes enriched by runs of homozygosity (ROH)

CHR	Positions (Mb)		% SNPs in ROH	Positional candiate genes
	Start	End		
Chr2	101.7	101.9	0.703	*STAM2*, *FMNL2*
	102.3	102.5	0.712	ENSBGRG00000007210
Chr3	21.8	22.0	0.703	*RFX5*, *SELENBP1*, ENSBGRG00000002099, *PSMB4*, *POGZ*
Chr4	127.8	128.0	0.723	None
	128.5	128.7	0.706	None
Chr5	80.0	80.2	0.733	*KRT82*, ENSBGRG00000000931, *KRT75*, ENSBGRG00000000991, ENSBGRG00000001018, ENSBGRG00000001074, ENSBGRG00000001103, *KRT71*
	81.0	81.2	0.732	*SP1*, ENSBGRG00000003334, *PCBP2*, *MAP3K12*, *TARBP2*, *NPFF*, *ATF7*
Chr6	55.4	55.6	0.733	*FRYL*, *ZAR1*, *SLC10A4*
	56.2	56.4	0.765	*CORIN*
	56.5	56.7	0.716	*CORIN*, *ATP10D*
Chr10	42.8	43.0	0.733	None
	43.5	43.7	0.721	None
Chr13	73.8	74.0	0.728	*SESN3*, ENSBGRG00000012109, ENSBGRG00000012137
	74.4	74.6	0.727	None
Chr27	28.6	28.9	0.733	*MICU1*, ENSBGRG00000024467, *MCU*

CHR: chromosomes

## Discussion

Rapid advances in high-throughput sequencing technologies have revolutionized the population genetics studies in model and non-model species [24]. In this study, we obtained genome-wide SNPs of yaks using DNA sequencing approach, by which the genetic differences between Maiwa and Jiulong yaks were revealed in terms of the allele frequency, nucleotide diversity, PCA-based sample clustering, and population differentiation index. Hence, our genomic data-based results are well consistent with the isolated geographical distribution and differentiated morphological characteristics of Maiwa and Jiulong yaks [25, 26]. However, the Fst differentiation index estimated between Maiwa and Jiulong yaks in this study was obviously lower than the previous estimates that were alternatively based on microsatellite DNA markers [27]. Wang et al. [14] analyzed genomic copy number variations and found that the studied samples of Maiwa and Jiulong yaks could not be fully separated from each other. Based on mitochondrial DNA variations, Maiwa and Jiulong yaks were revealed to be clustered into same matrilineal lineage [26, 28]. These results, on the other hand, may suggest that Maiwa and Jiulong yaks have the same genetic origin. Collectively, it seems that current genetic differences between Maiwa and Jiulong yaks would have been mainly resulted from the recent geographic isolation, as well as natural and artificial selection differentiation.

Villanueva et al. [29] and Caballero et al. [9] recently provided comprehensive comparisons among various estimators of genomic inbreeding using simulated or real data; one clear conclusion is that different estimators may have inconsistent and even contrasting estimates in both magnitude and direction. From a biological point of view, furthermore, the resulting ranges of estimates are hard to be explained for some of these estimators that are based on the expected allele frequency [29]. In this study, we similarly calculated four variant allele frequency-based estimators ($\:{F}_{L\&H}$, $\:{F}_{VR1}$, $\:{F}_{VR2}$, and $\:{F}_{YAN}$) for Maiwa and Jiulong yaks, and all of them provided consistent results that Maiwa yaks have the greater genomic inbreeding than Jiulong yaks. Sivalingam et al. [30] investigated genomic divergence among four Indian yak populations using genome-wide SNPs and reported the $\:{F}_{L\&H}$ varied from − 0.09 to 0.02, which are greater than our estimates in Maiwa and Jiulong yaks. Among the nine Chinese yak populations distributed in Qinghai, genomic inbreeding levels were measured by $\:{F}_{L\&H}$, with the highest and lowest values of 0.28 in wild yaks and − 0.04 in Huzhu white yaks, respectively [31]. The theoretical estimate of $\:{F}_{L\&H}$ is ranging from -∞ to 1, and negative values could be explained as that genetic variability has been gained during the past [29]. In this sense, Maiwa and Jiulong yaks have the greater genomic variability in comparison with other yak populations reported in literature. To our best knowledge, other three estimators of $\:{F}_{VR1}$, $\:{F}_{VR2}$, and $\:{F}_{YAN}$ have not been reported previously in literature for yaks.

In addition to allele frequency-based estimators, another more straightforward estimator is $\:{F}_{HOM}$ that does not depend on the expected allele frequencies in base population. In this study, our estimates of $\:{F}_{HOM}$ similarly supported that Maiwa yaks have the greater inbreeding. The observed $\:{F}_{HOM}$ in four Indian yak populations were comparable with or slightly lower than that in Maiwa yaks, but obviously higher than Jiulong yaks [30]. Li et al. [31] recently reported $\:{F}_{HOM}$ among nine Chinese yak populations, ranging from 0.65 in an indigenous population of Huzhu white yaks to 0.86 in the recently cultivated breed of Datong yaks; these values are comparable with or higher than our observation in Maiwa yaks. The bovine bone or leather remains can be usually found in archaeological and historical collections, which provide a rich source of historical DNA samples [32, 33]. Therefore, we collected a few old leather samples that may represent the historical Maiwa yaks about five generations ago, whereas only three of them were successfully sequenced for producing more 1000 clean SNPs. The smaller values of $\:{F}_{HOM}$ were observed among these leather samples, which indicate the lower inbreeding level. Of course, we need to interpret the results with much caution. First, only three leather samples were successfully analyzed, and their estimates of $\:{F}_{HOM}$ had a high variation. Second, historical generations of these samples were deduced according to farmers’ subjective declaration. Third, we have no direct evidence to support that these leather samples were exclusively derived from the same population of current Maiwa yaks.

Due to the independence on allele frequency and robustness to spatially variant noise, homozygous chromosome segment-based estimators have been proposed to provide more accurate estimation of genomic inbreeding in most cases [9]. The mean of $\:{F}_{ROH}$ estimates was 0.13 in Arunachali yak [34], which is much higher than our estimates in both Maiwa and Jiulong yaks. Despite of its independence on allele frequency, there are two possible limitations that prevent the direct comparison regarding the estimates of $\:{F}_{ROH}$ reported in different studies, including the subjective criteria to determine an effective ROH, and the varied density of genomic marker used [35, 36]. In this context, HBD segment-based estimation of genomic inbreeding employs some statistical models and hence does not strictly depend on the subjective criteria to determine the homozygous segments [11]. In contrast to the SNP-by-SNP estimators, our estimates of both $\:{F}_{ROH}$ and $\:{F}_{HBD}$ in this study revealed that there was no difference of inbreeding level between Maiwa and Jiulong yaks. Although ROH was scanned among nine Qinghai yak populations in literature [31], authors did not report the direct estimates of $\:{F}_{ROH}$. Furthermore, there was no obvious difference on both the number and length distribution of ROH identified between Maiwa and Jiulong yaks, while their overall lengths were slightly greater than the previous report in Arunachali yaks [34].

The geographically isolated populations may have the significantly differentiated genomic regions due to differences in the founder effect, environmental adaptation, artificial selection, and other demographic events [37]. Based on Fst differentiation index, we revealed the significantly differentiated genomic regions between Maiwa and Jiulong yaks. The positional candidate genes within these regions were significantly enriched into diverse molecular functions, such as endopeptidase, peptidase, hydrolase, and protein-macromolecule adaptor activity. However, it is impossible to make biological sense regarding these candidate genes if there is no specific context of population differentiation. As Jiulong yaks have the greater adult body size than Maiwa yaks [19], we further did literature search to determine whether some of these candidate genes are significantly associated with growth-related traits reported in livestock. Among them, *LPIN1* was reported to be significantly associated with milk production traits in dairy cattle [38, 39], and *MYOG*, *GREB1*, and *MYBPH* genes were involved in regulating the growth, carcass, and fertility traits in different farm animals [40–42]. Other genes, such as *TNNI3*, sphingomyelin phosphodiesterase 4 (*SMPD4*), and tubulin alpha 3d (*TUBA3D*) [43–45], were related to health traits in cattle. In the literature, however, no candidate gene was found to be associated with cardiac or pulmonary functions that may confer the physiological adaptation to high-altitude environments. In the future studies, these positional candidate genes revealed in this study may be specifically investigated regarding their differentiation and association with growth between Maiwa and Jiulong yaks.

Detection of ROH-enriched genomic regions provides a state-of-the-art approach for scanning genome-wide selection signatures [46]. As yaks have been raised mostly for dual purposes of milk and meat production, the underlying functional genes may be subjected to the artificial selection. In this study, we found that some positional candidate genes located within ROH-enriched regions have been previously reported to be significantly associated with diverse economically important traits in livestock, such as signal transducing adaptor molecule 2 (*STAM2*) and formin-like 2 (*FMNL2*) genes in relation to growth [47, 48], Keratin 75 (*KRT75*) and Keratin 71 (*KRT71*) genes affecting heat stress and health [49, 50], and mitogen-activated protein kinase kinase kinase 12 (*MAP3K12*), TARBP2 subunit of RISC loading complex (*TARBP2*), and Zygote arrest 1 (*ZAR1*) genes related to fertility [51–53]. For these positional candidate genes, however, specific investigations are required to reveal their associations with the production traits in yaks. Among the significantly enriched biological functions of candidate genes located within ROH-enriched regions in this study, the cellular components of intermediate filament and keratin filament were previously reported to be associated with the seasonal development dynamics of hair in yaks [54]. Also, the GO term of “intermediate filament cytoskeleton organization” was differed between fresh and frozen-thawed sperm of yaks [55]. We did not analyze the functional implications for these positional candidate genes that are located within these HBD regions as these chromosome segments are relatively short in length.

## Conclusions

We first evaluated the genomic inbreeding level, as well as genetic diversity, for two Chinese yak populations distributed in the southwest China, using genome-wide SNPs. Our comparisons revealed that Jiulong yaks have the greater genetic diversity than Maiwa yaks, but the lower or comparable genomic inbreeding level depending on which estimator is used. The results obtained in this study could be referred when implementing the genetic conservation and utilization programs for Maiwa and Jiulong yaks.

## Methods

### Sample collection and preparation of genomic DNA

The peripheral blood samples were collected by official veterinarians for a total of 73 Maiwa yaks and 30 Jiulong yaks, both of which are indigenous populations/breeds and locally distributed in Hongyuan County and Jiulong County of Sichuan Province, China, respectively. All Maiwa and Jiulong yaks were randomly collected among the adults that had been raised in 12 and five farms, respectively. Total genomic DNA was extracted using the standard procedure of the Animal Genomic DNA Kit (Tiangen, Beijing), and was evaluated for the quality and quantity using NanoVue Plus (GE, USA).

In the Hongyuan County where Maiwa yaks were geographically distributed, five old leather samples were successfully collected on the grain storage bags kept in different farmers, which were manufactured using the yak leather. These leather samples have the history of about 30 years according to farmers’ declaration, and therefore would represent the historical Maiwa yaks about five generations ago. To effectively extract genomic DNA from the largely degraded leather samples, we employed a combined method of guanidine hydrochloride lysis and phenol/chloroform extraction, as used in our previous study [56]. In brief, ~ 10 mg leather of each individual was sampled and rinsed in the distilled water overnight. Subsequently, the rinsed tissue was incubated using proteinase K (400 ng/mL) and guanidine hydrochloride (5 mol/L) solution in a total volume of 1 mL for 10 h at 55ºC. Finally, supernatant was collected after centrifugation at 12,000 g for 15 min, which was further subjected to the standard phenol/chloroform extraction.

### Genomic sequencing and genotyping

Genomic DNA was used for constructing paired-end libraries with 350 bp of insert sizes according to Illumina’s protocol (Illumina, San Diego, CA, USA). In brief, 0.5 µg of genomic DNA was fragmented, end-paired, and ligated to adaptors, respectively. After the P2 adapter was added, DNA fragments were fractionated and purified by PCR amplification. Finally, the qualified libraries were sequenced on Illumina HiSeq2000 at Biomarker Technology Company (Beijing, China).

The raw sequencing reads were subjected to quality controls using fastp software v0.23.4 [57], to discard the reads that contain low-quality bases (Qphred value < 5) more than 50% of the total length, ambiguous bases more than 10% of its total length, or adaptor sequence contamination. The clean reads were then mapped against the yak reference genome (BosGru3.1) using BWA-MEM algorithm in BWA software v0.7.17 with the default parameters [58]. SNP calling and genotyping were performed using GATK software v4.4.0.0 [59], according to recommendations of GATK Best Practices [60]. For the raw SNPs, we first performed hard filtering with expression of “QD < 2.0 || FS > 60.0 || MQ < 40.0 || MQRankSum < -12.5 || ReadPosRankSum < -8.0”. Subsequently, we retained biallelic autosomal SNPs that have the coverage depth of reads ≥ 6, and calling rate > 0.9 per variant and per individual. For the individual population of Maiwa or Jiulong yaks, SNPs were further discarded if they had the MAF < 0.01 or significant deviation from HWE (*P* < 10^− 6^). The calling rate, MAF, or HWE-based quality controls were not applied to these leather samples.

### Diversity and population structure

The genomic distribution of SNPs was visualized using circlize R package v0.4.16 [61]. Both MAF and nucleotide diversity were calculated for each of SNPs using vcftools software v0.1.16 [62]. To demonstrate individual relatedness and population structure, PCA was performed using SNPRelate R package v1.36.1 [63], to which the tightly linked SNPs (r^2^ > 0.9) were excluded in advance. Within each population, average genetic relatedness among individuals was calculated using GCTA software v1.94.1 with the default parameters [64]. The historical Ne of Maiwa and Jiulong yaks were deduced using genome-wide SNPs and SNeP software v1.111, which addressed some possible issues in relation to sample size, mutation, phasing, and recombination rate [65]. Using vcftools software v0.1.16 [62], Weir and Cockerham’s Fst between Maiwa and Jiulong yaks was computed for each chromosome window, whose size and sliding step were set to 200 Kb and 100 Kb, respectively. The significantly differentiated window was defined if its Fst was deviated by more than 3.5 SD from the mean across genome.

### Genomic inbreeding estimators

Five SNP-by-SNP estimators of genomic inbreeding were calculated separately for Maiwa and Jiulong yaks. Following Villanueva et al. [29] and Caballero et al. [9], two homozygosity derivation-based estimators were first calculated, including the simple proportion of homozygous SNPs ($\:{F}_{HOM}$) and its adjusted proportion by the expected allele frequencies ($\:{F}_{L\&H}$), as follow:

$$\:{F}_{HOM}=1-\frac{{\sum\:}_{k=1}^{S}{x}_{k}\left(2-{x}_{k}\right)}{S},$$

where $\:S$ is the total number of SNPs; $\:{x}_{k}$ is the individual genotype of SNP $\:k$ coded as 0, 1, or 2 (i.e., representing the number of the B allele). When the expected frequency of B allele ($\:{p}_{k}$) is available, the adjusted proportion of homozygous SNPs could be derived as [66]:

$$\:{F}_{L\&H}=1-\frac{{\sum\:}_{k=1}^{S}{x}_{k}\left(2-{x}_{k}\right)}{{\sum\:}_{k=1}^{S}2{p}_{k}\left(1-{p}_{k}\right)},$$

Here, $\:{p}_{k}$ was set to the observed frequencies in their respective current populations of Maiwa and Jiulong yaks. Genomic relationship matrix has been wildly used in the genomic evaluation [67], and individual inbreeding coefficient can be obtained from its diagonal elements. As proposed by VanRaden et al. [68], two similar estimators of $\:{F}_{VR1}$ and $\:{F}_{VR2}$ were calculated as:

$$\:{F}_{VR1}=\frac{{\sum\:}_{k=1}^{S}{({x}_{k}-2{p}_{k})}^{2}}{{\sum\:}_{k=1}^{S}2{p}_{k}(1-{p}_{k})}-1,\:\text{a}\text{n}\text{d}$$

$$\:{F}_{VR2}=\frac{1}{S}\sum\:_{k=1}^{S}\left(\frac{{\left({x}_{k}-2{p}_{k}\right)}^{2}}{2{p}_{k}\left(1-{p}_{k}\right)}-1\right),\:\text{r}\text{e}\text{s}\text{p}\text{e}\text{c}\text{t}\text{i}\text{v}\text{e}\text{l}\text{y}.$$

Based on correlation between uniting gametes, individual inbreeding coefficient could be alternatively calculated as [69]:

$$\:{F}_{YAN}=\frac{1}{S}\sum\:_{k=1}^{S}\frac{{x}_{k}^{2}-\left(1+2{p}_{k}\right){x}_{k}+2{p}_{k}^{2}}{2{p}_{k}\left(1-{p}_{k}\right)}.$$

After excluding the tightly linked SNPs ($\:{r}^{2}$ > 0.9), we calculated the estimators of $\:{F}_{HOM}$, $\:{F}_{L\&H}$, $\:{F}_{VR2}$, and $\:{F}_{YAN}$ using plink software v1.9 [70], and $\:{F}_{VR1}$ using GCTA software v1.94.1 [64]. Because of small sample size, only $\:{F}_{HOM}$ was applied to three old leather samples that have more than 1000 clean SNPs.

In addition to these SNP-by-SNP estimators above, genomic inbreeding could be measured according to genomic distribution of ROH [10]. We similarly excluded the tightly linked SNPs ($\:{r}^{2}$ > 0.9) and scanned the genome-wide ROH for Maiwa and Jiulong yaks using detectRUNS R package v0.9.6 [71], in which a window of 15 SNPs was slid across genome using a step size of one SNP. No more than one missing SNP and one SNP with opposite genotype was permitted in each window, respectively. An effective ROH was defined by requiring at least 20 SNPs in total, minimum length of 500 Kb, and minimum density of one SNP per 200 Kb. The parameters of minimum number of SNPs, minimum length, and minimum density required for an effective ROH were empirically determined in this study, while other parameters were set by the default values. Herein, ROH-based genomic inbreeding of $\:{F}_{ROH}$ could be calculated as:

$$\:{F}_{ROH}=\frac{\sum\:{L}_{ROH}}{{L}_{AUTO}},$$

where $\:\sum\:{L}_{ROH}$ is the cumulative length across all ROH detected; $\:{L}_{AUTO}$ is the total autosomal length covered by SNPs.

Following Lozada-Soto et al. [11], we further employed a model-based approach to find HBD chromosome segments using RZooRoH R package v0.3.2.1 [72], which treats that the length of HBD segments is exponentially distributed, and estimates the probability when including a SNP into a larger HBD segment through hidden Markov model. Similarly [11], we set seven length classes of HBD segments in this study, which had the rates of the exponential distribution of $\:{2}^{\text{n}}$ (*n* = 1–7), respectively. The HBD-based genomic inbreeding of $\:{F}_{HBD}$ was measured by summing the estimates across all the seven HBD classes.

### Functional analyses of candidate genes

Within these genomic regions that were indicated by the significant Fst or enriched by ROH, we extracted the positional protein-encoding and lncRNA genes using biomaRt R package v2.58.2 [73]. Subsequently, functional enrichment analyses were performed using gprofiler2 R package v0.2.3 [74], with the target datasets of the GO biological knowledge [75]. The default g: SCS approach of multiple testing correction was used for computing the adjusted *P* values for gene enrichments, and a threshold of 0.05 was set.

### Electronic supplementary material

Below is the link to the electronic supplementary material.

Supplementary Material 1

## Data Availability

The raw sequence data reported in this paper have been deposited in the Genome Sequence Archive of Chinese National Genomics Data Center (GSA: CRA015178) that are publicly accessible at https://ngdc.cncb.ac.cn/gsa.

## Abbreviations

Fst: Population differentiation index
GO: Gene Ontology
HBD: Homozygous-by-descent
LD: Linkage disequilibrium
lncRNA: Long non-coding RNA
MAF: Minor allele frequency
PCA: Principal component analysis
Ne: Effective population sizes
ROH: Runs of homozygosity
SD: Standard deviation
SNP: Single nucleotide polymorphism
